# Retrospective Evaluation of Early Glargine Administration in Adults With Diabetic Ketoacidosis

**DOI:** 10.7759/cureus.90566

**Published:** 2025-08-20

**Authors:** Christopher Lesniak, Paula Gonzalez Espinosa, Rosily Philip, Farah Farhat, Eram Ashan, Minhaz Murshad, Ashna Gupta, Mohammad A Hossain, Jennifer Cheng, Raquel Ong, Krishna Chalasani, Katherine Hu, Monika Akula, Soemiwati Holland

**Affiliations:** 1 Endocrinology, Diabetes and Metabolism, Jersey Shore University Medical Center, Neptune, USA; 2 Internal Medicine, Jersey Shore University Medical Center, Neptune, USA; 3 Medicine, Hackensack Meridian School of Medicine, Nutley, USA; 4 Endocrinology and Diabetes, Jersey Shore University Medical Center, Neptune, USA

**Keywords:** diabetes, glargine, insulin, ketoacidosis, medical icu

## Abstract

Purpose

Diabetic ketoacidosis (DKA) is a life-threatening condition, and treatment consists of aggressive fluid replacement and correcting the insulin deficit to resolve the acidosis and hyperglycemia. American Diabetes Association (ADA) guidelines recommend administering insulin glargine after resolution. The purpose of this retrospective study was to determine if early glargine, given within six hours of starting insulin infusion, decreased insulin infusion time in patients admitted to the hospital with DKA.

Methods

Patients admitted to our hospital in 2021 with a diagnosis of DKA who were treated with an insulin drip were analyzed. Rebound hyperglycemia was analyzed using Pearson’s chi-squared test. Stepwise linear regression analysis was performed for the primary outcome, insulin duration, and the main predictor was early versus late glargine.

Results

Duration of insulin infusion was shorter in the early glargine group (p=.043). There was a higher incidence of rebound hyperglycemia (>180 mg/dL) in the six hours after insulin discontinuation in the late glargine group, 63 (83%) vs. 35 (50%) (p<0.001). An interaction between early glargine and type 1 diabetes was significant (p=0.047), with analysis showing that early glargine did not reduce the infusion duration in type 1 diabetes but significantly reduced the duration in patients without type 1 diabetes (p=0.010).

Conclusion

Early glargine administration can decrease morbidity from rebound hyperglycemia and reduce the cost of care by minimizing the amount of time required to be on an insulin drip in a critical care setting. This effect was only found in individuals without type 1 diabetes, which may require further study.

## Introduction

Diabetic ketoacidosis (DKA) is a preventable, yet life-threatening complication of uncontrolled hyperglycemia and diabetes mellitus. DKA consists of a triad of hyperglycemia, metabolic acidosis, and increased blood ketones that results from inadequate insulin levels, leading to inappropriate metabolism of triglycerides and amino acids. Definitions for DKA generally include glucose > 200 mg/dL, pH < 7.3, and bicarbonate < 18 mmol/L, with detectable urinary or serum ketones [1].

Treatment for DKA consists of aggressive fluid replacement and correction of the insulin deficit to help resolve the acidosis and hyperglycemia [1]. Many institutions have standardized protocols ranging from weight-based insulin infusions to titratable nursing protocols. There is a general consensus that resolution of DKA includes glucose < 200 mg/dL and two of the following: serum bicarbonate level ≥ 18 mEq/L, venous pH > 7.3, and calculated anion gap ≤ 12 mEq/L [1]. At the time of resolution, there needs to be an overlap in the administration of a long-acting insulin and the stopping of the insulin infusion of 1-2 hours, due to the onset of action of long-acting insulin analogues. 

Despite improvements with standardized treatment protocols, DKA remains an emergent condition that can cause significant morbidity and mortality if left untreated, with a recent study finding 0.4% mortality. In a recent report, the trend of cases of DKA increased 13.5% annually from 2009 to 2015 [2]. This increase was evident in all age groups among both men and women in most regions of the country. Infection is a common trigger for DKA, and these numbers are likely higher now after the COVID-19 pandemic [3]. 

Similarly, as the number of cases of DKA has increased, so has the burden on the US healthcare system. Despite improvements in treatment and decreases in the length of stay, costs have continued to rise from $2.2 billion in 2003 up to $5.1 billion. Driving forces of this cost are the time spent in intensive care on IV insulin infusion, which requires closer nursing care and hourly blood glucose checks, titration of insulin, and fluids [4].

Insulin glargine is a long-acting insulin analogue that has shown multiple improvements over neutral protamine hagedorn (NPH) insulin, including greater metabolic activity, longer duration of action, and a peakless effect. The pharmacokinetics and pharmacodynamics result in a close approximation of the gold standard for basal insulin replacement, which is continuous subcutaneous insulin infusion [4]. Transitioning to long-acting insulin for a 1-2 hour overlap at the time of DKA resolution has decreased the glycemic variability between intravenous and subcutaneous injections of insulin and reduced episodes of rebound hyperglycemia [5]. 

There have been multiple studies in both pediatric and adult populations that have looked at the administration of insulin glargine earlier in the course of DKA prior to resolution, with mixed results [6-15]. Several studies have suggested that there is no increase in harm through hypoglycemia with the addition of insulin glargine [6,9-11,13-14]. Although individual results differ, studies have shown improvements in time to resolution of DKA, insulin infusion time, intensive care unit (ICU) length of stay, hospital length of stay, and rebound hyperglycemia after the insulin infusion is stopped [7-8,10,12,14]. Although there is no definitive consensus among societies, the Joint British Diabetes Societies guidelines include the continuation of long-acting insulin analogues during the initial management of DKA [16]. Conversely, the ADA only recommends using basal insulin during the transition period between infusion and subcutaneous injections [1]. 

The goal of this study was to determine if early insulin glargine administration (within six hours of starting insulin infusion) decreased the duration of insulin infusions and hospital stay versus the standard administration of insulin glargine in patients admitted with diabetic ketoacidosis. Secondary outcomes included length of hospital stay and rebound hyperglycemia, defined as a glucose measure of > 180 mg/dL in the six hours after the insulin drip was stopped.

## Materials and methods

This is a single-hospital retrospective analysis of adult patients diagnosed with DKA at Jersey Shore University Medical Center from the period of January 1, 2021, and December 31, 2021. Inclusion criteria were age greater than or equal to 18 years of age and patients who were diagnosed on the criteria of glucose > 250 with pH < 7.30, bicarbonate < 18 mEq/L, and anion gap > 12, who were placed on an insulin infusion. Exclusion criteria included patients less than 18 years old, patients who were pregnant, patients on end-of-life care and/or hospice, and patients diagnosed with DKA but who were never administered insulin infusion. Approval for this study was granted by the Hackensack Meridian Health Institutional Review Board, IRB ID# Pro2020-1093. Additionally, a waiver of consent and a waiver of Health Insurance Portability and Accountability Act (HIPAA) authorization were granted for this study. 

Descriptive statistics

Descriptive statistics were used to summarize demographic information, inpatient management, and initial laboratory values. Continuous variables were summarized with means (standard deviations) or medians (interquartile ranges). Categorical variables were summarized with counts and percentages. Normality and equality of variance in outcome variables were tested with the Shapiro-Wilk test and the Fligner-Killeen test, respectively. Because the normality assumption was not satisfied, a non-parametric Wilcoxon rank sum test was used to test for location shift (medians) in outcomes between the two groups.

Multiple linear regression

To adjust for the possible influence of covariates, linear regression analysis was performed. The primary outcome was the duration of insulin (log-transformed, base), and the main predictor of interest was the group membership (Glargine administration: early vs. late). We used stepwise regression to choose the best model based on AIC (Akaike’s information criteria) [17]. This model contained main effects and interactions. Model diagnostics were performed using various diagnostic plots. Based on the diagnostics, three patients were determined to be outliers and had a large influence on the regression. These patients were removed, and the model was refit.

Software

R Foundation for Statistical Computing, Vienna, Austria, was used for all analysis [17]. Stepwise regression was implemented with the stepAIC function from the Medical Aids Subsidy Scheme (MASS) package [18].

## Results

From January 1, 2021, to December 31, 2021, there were 146 patients diagnosed with DKA who met the criteria for inclusion in the study. Baseline data for continuous and categorical variables of our study population are included in Table 1. The median age of our population was 52 years (IQR: 35-66). Our population consisted of 75 (51%) females and 72 (49%) males. We had 57 patients (39%) with type 1 diabetes and 80 patients (55%) with type 2 diabetes. Nine patients (6%) did not have a previous diagnosis of diabetes.

**Table 1:** Baseline characteristics IQR: Interquartile range, HTN: Hypertension, HLD: Hyperlipidemia, CKD: Chronic kidney disease, CAD: Coronary artery disease, T2DM: Type 2 diabetes mellitus, T1DM: Type 1 diabetes mellitus, BMI: Body mass index

Characteristic	Overall, N=146	Late Glargine, N=76	Early Glargine, N=70	Group Comparison	P-value
Age, years, median (IQR)	52 (35-66)	51 (34-63)	53 (36-71)	W = 2,315	0.18
Ethnicity, n (%)				χ^2^ = 4.1	0.13
Hispanic or Latino	15 (10)	7 (9.2)	8 (11)		
Not Hispanic or Latino	112 (77)	55 (72)	57 (81)		
Unreported	19 (13)	14 (18)	5 (7.1)		
Race, n (%)					0.14
Asian	2 (1.4)	0 (0)	2 (2.9)		
Black	31 (21)	14 (18)	17 (24)		
Caucasian	82 (56)	41 (54)	41 (59)		
More than One Race	2 (1.4)	1 (1.3)	1 (1.4)		
Unreported	29 (20)	20 (26)	9 (13)		
Gender, n (%)				χ^2^ = 0.70	0.40
Female	74 (51)	36 (47)	38 (54)		
Male	72 (49)	40 (53)	32 (46)		
HTN, n (%)	72 (49)	33 (43)	39 (56)	χ^2^ = 2.2	0.14
HLD, n (%)	71 (49)	35 (46)	35 (51)	χ^2^ = 0.42	0.52
CKD, n (%)	15 (10)	6 (7.9)	9 (13)	χ^2^ = 0.97	0.32
CAD, n (%)	18 (12)	8 (11)	10 (10)	χ^2^ = 0.48	0.49
T2DM, n (%)	80 (55)	37 (49)	43 (61)	χ^2^ = 2.4	0.12
T1DM, n (%)	57 (39)	34 (45)	23 (33)	χ^2^ = 2.2	0.14
BMI, median (IQR)	27 (23-32)	27 (23-32)	26 (23-32)	W = 2,774	0.66

At the time of diagnosis, median HbA1c was 11.1 (IQR: 9.2-13.5). The median glucose was 570 (IQR: 424-727), and the anion gap was 22 (IQR: 19.0-26.2). There was a small but statistically significant difference in the pH at diagnosis of patients who received early glargine (median: 7.20, IQR: 7.09-7.29) compared to 7.24 (IQR: 7.18-7.31) in those who did not receive glargine (p=0.046). Baseline laboratory values are in Table 2.

**Table 2:** Baseline laboratory values *p<0.005 IQR: Interquartile range, HbA1C: Hemoglobin A1C

Characteristic	Overall, N=146	Late Glargine, N=76	Early Glargine, N=70	Group Comparison	P-value
HbA1c, median (IQR)	11.10 (9.23-13.45)	11.10 (9.35-13.53)	11.10 (9.23-13.23)	W=2,634	0.92
pH, median (IQR)	7.23 (7.13-7.30)	7.24 (7.18-7.31)	7.20 (7.09-7.29)	W=3,047	0.046*
Glucose (mg/dL), median (IQR)	570 (424-727)	564 (425-716)	572 (424-751)	W=2,575	0.74
Creatinine mg/dL, median (IQR)	1.68 (1.30-2.13)	1.61 (1.30-2.11)	1.69 (1.30-2.14)	W=2,624	0.89
Beta-hydroxybutyrate mmol/L, median (IQR)	6.10(3.59-8.00)	6.74 (3.57-8.00)	5.83 (3.64-8.00)	W=2,519	0.65
Bicarbonate mmol/L, median (IQR)	14.0 (19.0-26.2)	22.4 (19.0-26.3)	21.5 (18.3-26.2)	W=2,943	0.27
Anion Gap mmol/L, median (IQR)	22.0 (19.0-26.2)	22.4 (19.0-26.3)	21.5 (18.5-26.2)	W=2,672	0.97

The independent variable being measured in this study was the exposure to glargine within the first six hours of insulin infusion initiation. As shown in Table 3, 70 patients received glargine within the first six hours, and 76 did not. Those who received early glargine had a statistically significant decrease in the length of infusion, with a median infusion time of 11 hours (IQR: 6-20), compared to 14 (IQR: 8-30) hours in those who did not receive early glargine (Wilcoxon rank sum test, p=0.043). Rebound hyperglycemia was defined as glucose greater than 180 mg/dL within the first six hours after discontinuation of insulin infusion. Fifty percent of patients (35/70) who received early glargine had rebound hyperglycemia compared to 63% of patients (63/76) who did not receive early glargine (Pearson’s Chi-squared test, p < 0.001). There was no difference in hospital length of stay between these two groups, with a median length of stay of five (IQR: 5-8) days (p=0.94).

**Table 3:** Primary and secondary outcomes *p<0.05 IQR: Interquartile range, SD: Standard deviation

Characteristic	Overall, N=146	Late Glargine, N=76	Early Glargine, N=70	Group Comparison	P-value
Length of stay, days				W=2,679	0.94
Mean (SD)	7 (7)	7 (6)	7 (8)		
Median (IQR)	5 (3, 8)	5 (3, 8)	5 (3, 8)		
Minimum – Maximum	1-44	1-39	1-44		
Length of Infusion, hours				W=3,176	0.043*
Mean (SD)	18 (16)	20 (17)	16 (14)		
Median (IQR)	12 (7, 25)	14 (8, 30)	11 (6, 20)		
Minimum – Maximum	1-96	1-96	2-70		
Hyperglycemia, n (%)	98 (67)	63 (83)	35 (50)	χ^2^ = 18	<0.001*

Stepwise regression in both directions was performed with the initial model that contained all main effects and interactions between the glargine variable and comorbidities using a full dataset. Stepwise regression resulted in a final model that had the smallest Akaike Information Criterion (AIC). Diagnostics of the final model indicated that three patients appeared to be outliers and had a large influence on the regression. These patients were removed from the dataset, and the final model was refitted. The model used is shown in Table 4.

**Table 4:** Linear regression model table aDiagnostics of the final model indicated that three patients appeared to be outliers and had a large influence on regression. These patients were removed from the dataset, and the final model was refit. HTN: Hypertension, HLD: Hyperlipidemia, CKD: Chronic kidney disease, CAD: Coronary artery disease, T2DM: Type 2 diabetes mellitus, T1DM: Type 1 diabetes mellitus, BMI: Body mass index

Variables	All Data				Three patients removed^a^			
	Estimate	Lower 95% CI	Upper 95% CI	P-value	Estimate	Lower 95% CI	Upper 95% CI	P-value
Intercept	10.75	4.29	26.91	0.000	8.26	3.62	18.86	0.000
Glargine < 6 hours	-1.37	-3.16	-1.23	0.005	-1.76	-2.70	-1.14	0.010
Ethnicity Not Hispanic	-1.27	-01.94	1.20	0.264	-1.55	-2.29	-1.05	0.029
Ethnicity Unreported	-1.79	-3.08	-1.05	0.034	-1.84	-3.03	-1.12	0.016
HTN	-1.67	-2.47	-1.13	0.011	-1.60	-2.29	-1.12	0.010
HLD	1.36	-1.02	1.88	0.065	1.38	1.02	1.86	0.035
CKD	-1.44	-2.28	1.10	0.116	-1.75	-2.66	-1.15	0.009
CAD	-1.44	-2.12	1.06	0.095	-1.61	-2.33	-1.11	0.012
T2DM	1.42	-1.17	2.35	0.173	1.54	-1.02	2.42	0.063
T1DM	1.24	-1.41	2.17	0.451	1.35	-1.22	2.23	0.233
BMI	1.02	-1.00	1.04	0.086	1.03	1.01	1.05	0.002
Glargine <6 hours x HTN	1.59	-1.08	2.74	0.092	1.51	-1.09	2.48	0.105
Glargine <6 hours x T1DM	1.76	-1.01	3.11	0.054	1.68	1.01	2.81	0.047

Based on the regression model, receiving early glargine decreased the insulin infusion time by a factor of 1.76 (p=0.010) when controlling for demographics and comorbidities (Table 4). BMI had a modest effect on insulin duration. An increase of BMI by one unit increased the infusion time by a factor of 1.03.

Interestingly, there was an interaction noted between those with type 1 diabetes mellitus and early glargine administration (p=0.047). Therefore, the effect of glargine was examined separately for patients who had type 1 diabetes mellitus and those who did not (Table 5). Results are shown in Table 5. When type 1 diabetes mellitus was present, there was no significant reduction of infusion time between the glargine groups (p>0.999). In patients without type 1 diabetes mellitus, early administration of glargine significantly affected the length of infusion (p=0.021), adjusting for other variables in the model. In those without type 1 diabetes mellitus, the model predicts that, on average, early administration of glargine will reduce the infusion time by a factor of 1.76 (95% CI of the difference: -2.70 to -1.14).

**Table 5:** Effect of type 1 diabetes mellitus on the relationship between early glargine and length of infusion *p<0.05 T1DM: Type 1 diabetes mellitus

Comorbidity	Glargine < 6 hours	Predicted	Lower 95% CI	Upper 95% CI	Reduction Factor	Lower 95% CI	Upper 95% CI	P-value
T1DM								
No	No	11.94	7.51	18.97	1.76	1.14	2.7	0.021*
	Yes	6.79	4.06	11.36				
Yes	No	16.16	12.17	21.47	1.04	0.7	1.56	>0.999
	Yes	15.49	11.40	21.05				

## Discussion

The purpose of this retrospective study was to determine the difference between early versus late glargine administration and insulin infusion time among patients admitted to the hospital with DKA. The results show a significant decrease in the length of insulin infusion in the group that received early glargine. Secondary endpoints analyzed show fewer episodes of rebound hyperglycemia after termination of the insulin infusion. Surprisingly, despite the decrease in duration of infusion, there was no significant decrease in length of stay among our population. Previous studies [6-15] have shown some benefit and little harm in the early administration of insulin glargine in DKA, although definitions of early glargine doses are not homogeneous. There was also an interaction between patients with type 1 diabetes mellitus and the main outcome, which, when further analyzed, did not show any benefit of early glargine compared to those without type 1 diabetes.

While more common in type 1 diabetes, DKA can also occur in ketosis-prone type 2 diabetes. In fact, there has been an increasing number of cases among those with type 2 diabetes. It was previously believed that because type 2 diabetes is a function of insulin resistance, there was still adequate insulin secretion from the pancreas to prevent ketone buildup. Usually, there is a precipitating factor such as infection, surgery, poor medication compliance, or a cardiovascular event that creates a relative insulin deficiency, leading to DKA [19].

In a prospective randomized trial, patients received insulin glargine at a standard 0.25 units/kg of body weight within the first 12 hours of starting infusion therapy. Although this study did not show any significant difference in infusion time, patients in the early glargine group had a lower rate of hyperglycemia (>180 mg/dL) within the first 12 hours after the infusion was stopped without any increased rates of hypoglycemia [9]. 

In a 2015 randomized control trial, early glargine was given in the first three hours after starting the insulin infusion, and although it did not show any significant differences, the authors reported a trend of decreased time to anion gap closure in the early glargine group. The failure to reach statistical significance was attributed to the sample size of the population. They did show a reduced rate of rebound hyperglycemia. Significantly, compared to our study, this population was heavily favored toward patients with type 1 diabetes mellitus, and the ineffectiveness of early glargine in this population may have contributed to the lack of significant difference in infusion time [13]. 

In a 2015 study by Doshi et al., early glargine, defined as within two hours of diagnosis, was examined against intravenous insulin only. The study did not find any significant differences in time to anion gap closure or length of stay but did note a trend in the findings that did not reach statistical significance, presumably due to being underpowered [6]. 

A 2019 retrospective cohort study by Rappaport et al., with early glargine defined as before resolution of DKA vs. after DKA resolution, showed a significantly shorter time to correction of acidosis as well as a shorter time of infusion. There was a trend towards shorter hospital stays that did not reach significance [8].

A 2021 retrospective cohort study examining the correlation between time to glargine administration and time to DKA resolution showed that for every six-hour delay in the administration of glargine, there was a significant increase in infusion duration, intensive care unit length of stay, hospital length of stay, incidence of hypokalemia, and incidence of hypoglycemia (<80 mg/dL) [10]. 

However, a retrospective analysis by Kinney et al. showed that early glargine, defined as at least four hours before the infusion was stopped, was associated with an increased time on insulin infusion without any other measures reaching significance [15]. This definition of four hours before infusion was stopped is closer to the standard ADA recommendation of 1 to 2 hours and may not be comparable with the other studies, in which the definition of early glargine was considered to be closer to diagnosis or the beginning of intravenous insulin, prior to drip termination.

Studies in the pediatric population have shown similarly mixed results, with two studies showing early glargine associated with decreased insulin infusion duration and DKA resolution [7,12], while another did not show differences aside from increased rates of hypokalemia in the early glargine group [11]. All of these studies consisted entirely of pediatric patients with type 1 diabetes mellitus, so the results are not entirely comparable to studies in adults.

A recent 2023 prospective randomized controlled trial evaluated insulin glargine plus intravenous insulin versus intravenous insulin alone. This study used a standard 0.3 units/kg within the first three hours of diagnosis. This study showed a faster resolution of DKA, as well as a shorter length of stay for those who received glargine. This patient population was heavily weighted with 42 (76.7%) patients with type 2 diabetes, compared to our study, which consisted of 80 (55%) patients with known type 2 diabetes. In their subgroup analysis, they reported an even bigger difference in the time to DKA resolution among those with type 2 diabetes than the whole study population, which may align with our interaction between type of diabetes and outcomes, although they did not present the findings in this manner [14]. 

A proposed mechanism for the benefit of additional glargine in type 2 patients that was not present in type 1 patients may be related to insulin resistance. Initial DKA in patients with type 2 diabetes usually occurs in the setting of long-standing hyperglycemia and additional stressors that increase counter-regulatory hormones. These hormones can reduce insulin secretion and downregulate glucose transporters and insulin gene transcription. Patients with type 2 diabetes tend to be older and have other features of insulin resistance, such as obesity, that may necessitate larger amounts of insulin to correct the acidosis [16]. Once glargine is given subcutaneously, it would continue to affect glucose transport independent of the titration of intravenous insulin. This may help overcome resistance quicker and lead to decreased time spent on insulin infusion and faster resolution of ketosis in these patients.

This study’s strengths included a real-world population of patients with diabetic ketoacidosis. The population reflected the increasing trend of patients with type 2 diabetes mellitus who experience episodes of DKA, including 80 (55%) patients in this study. Tangible benefits can be seen in the outcome that would translate to less time required in the ICU, minimizing costs, as well as a reduced rate of rebound hyperglycemia, likely adding to patient safety and possibly reducing relapses into DKA. The major weaknesses of this study are twofold. First, due to the retrospective nature of this study, if providers administered insulin glargine early in the disease course, the dose was left to their discretion, allowing for provider bias. This creates variation in the amount of insulin glargine given in the intervention group. Second, because of the retrospective nature, it was unclear when the diagnosis of DKA was actually made, and instead, the early glargine cut-off was made within six hours of starting the insulin drip. There is a possibility of delays in care in starting the insulin drip due to bed management, as patients in this institution are either treated in the medical intensive care unit or housed in the emergency department until a bed opens.

## Conclusions

Our findings agree with previous studies and add to the evidence to suggest some benefits and minimal risk of adding subcutaneous glargine early in the treatment of DKA in addition to standard intravenous insulin therapy. This aligns with guidelines by the Joint British Diabetes Societies, although this is not standard of care from the American Diabetes Association. While our study does show an interaction where the effect of glargine is more pronounced in patients with type 2 diabetes over type 1 diabetes, there does not seem to be any harm in initiating early glargine for type 1 patients. Larger prospective studies may be able to further delineate the benefit and provide reasonable evidence to update future guidelines.
